# A Matcha Tea Formulation Containing *Lachancea thermotolerans*: Phytochemical Profile After In Vitro Digestion and Effects on Intestinal Epithelial Function

**DOI:** 10.3390/nu18183004

**Published:** 2026-09-14

**Authors:** Chiara Rucci, Giulia Feliziani, Alice Agarbati, Francesca Comitini, Sauro Vittori, Cinzia Mannozzi, Riccardo Marconi, Laura Bordoni, Rosita Gabbianelli

**Affiliations:** 1 Unit of Molecular Biology and Nutrigenomics, School of Pharmacy, University of Camerino, 62032 Camerino, Italy; chiara.rucci@unicam.it (C.R.); giulia.feliziani@unicam.it (G.F.); laura.bordoni@unicam.it (L.B.); 2 School of Advanced Studies, University of Camerino, 62032 Camerino, Italy; 3 Department of Life and Environmental Sciences, Università Politecnica delle Marche, 60131 Ancona, Italy; a.agarbati@univpm.it (A.A.); f.comitini@univpm.it (F.C.); 4 Chemistry Interdisciplinary Project (CHIP), School of Pharmacy, University of Camerino, 62032 Camerino, Italy; sauro.vittori@unicam.it (S.V.); cinzia.mannozzi@unicam.it (C.M.); riccardo02.marconi@unicam.it (R.M.)

**Keywords:** matcha, probiotic yeast, antioxidant, functional food, *Lachancea thermotolerans*, anti-inflammatory potential

## Abstract

Background/Objectives: Matcha tea is widely recognized for its antioxidant and anti-inflammatory properties; however, its health-promoting benefits depend on the gastrointestinal bioaccessibility of its phytochemicals. This study evaluated the impact of combining matcha tea with the probiotic yeast *Lachancea thermotolerans* DiSVA322 on bioactive stability and intestinal epithelial protection. Methods: Formulations of matcha tea alone (M), matcha tea combined with the probiotic yeast (M + P), and the probiotic alone (P) were subjected to in vitro gastrointestinal digestion. Probiotic survival, total antioxidant capacity, and polyphenol profiles were analyzed. The biological effects of the digested fractions were tested on inflamed Caco-2 cell monolayers to assess epithelial barrier integrity and gene expression profiles. Results: The digested M + P formulation exhibited a significantly higher polyphenol content compared to M alone, including a 322% increase in gallic acid alongside a 40.5% reduction in caffeine content. Both M and M + P digests protected the intestinal barrier against inflammatory damage, preventing the decline in TEER values. However, only the M + P formulation successfully mitigated the compensatory upregulation of the Claudin-1 gene, while significantly downregulating the pro-inflammatory transcription factor NF-κB and the mitochondrial antioxidant SOD2. Furthermore, *L. thermotolerans* DiSVA322 demonstrated significantly higher survival when co-ingested with Matcha tea (35.15% viability) compared to mineral water (14.95% viability). Conclusions: Enriching matcha tea with the probiotic yeast *L. thermotolerans* DiSVA322 improves its chemical composition: the tea matrix serves as a protective vehicle that enhances probiotic survival, while the yeast modulates and optimizes the phytochemical bioactive profile and enhances the nutrigenomic responses in the intestinal mucosa.

## 1. Introduction

Tea is the second most consumed beverage worldwide after water and has long been appreciated not only for its sensory properties but also for its health-promoting effects [1,2]. Epidemiological and experimental studies indicate that regular tea consumption is associated with antioxidant, anti-inflammatory, lipid-lowering, and chronic disease-preventive effects, which have been mainly attributed to its rich phytochemical composition [3,4,5]. Among the different types of green tea, matcha has attracted increasing scientific and commercial interest over the past decade owing to its unique production process and exceptionally high concentration of bioactive compounds [6]. Unlike traditional green tea infusions, matcha is consumed as a fine powder dispersed in water, allowing the ingestion of the entire leaf and consequently a higher intake of bioactive constituents [7]. The distinctive phytochemical profile of matcha is largely attributed to its cultivation process, which involves shading the *Camellia sinensis* plants for several weeks before harvest [7].

Matcha contains a wide range of bioactive compounds, including catechins, flavonols, phenolic acids, chlorophylls, and amino acids, which collectively contribute to its biological activity [7]. Among these, epigallocatechin gallate (EGCG) is the predominant catechin and has received particular attention because of its potent antioxidant and anti-inflammatory properties [6,8]. However, the biological effects of matcha cannot be attributed to a single phytochemical. Increasing evidence suggests that flavonols, phenolic acids, and other minor constituents interact through additive or synergistic mechanisms, collectively contributing to the health-promoting properties of matcha. This complex phytochemical profile has therefore positioned matcha as a promising functional food for preventing or mitigating oxidative stress- and inflammation-related disorders [4].

Nevertheless, these biological effects depend not only on the composition of matcha tea, but also on the fraction of bioactive compounds that becomes bioaccessible during gastrointestinal digestion [9]. Indeed, the food matrix and the interactions among its phytochemicals influence their stability, release, and bioaccessibility during digestion [9]. Therefore, investigating digested matcha provides a more physiologically relevant approach to evaluate its biological activity than studying the native product alone [9]. For this reason, evaluating the bioaccessible fraction represents a more physiologically relevant strategy for investigating the health effects of matcha than relying solely on the native product [9,10]. As the first interface between dietary compounds and the human body, the intestinal epithelium plays a central role in nutrient absorption, barrier integrity, immune regulation, and redox homeostasis. Preserving epithelial function is particularly important under inflammatory conditions, where oxidative stress and tight junction disruption contribute to increased intestinal permeability and disease progression [11].

To further enhance the beneficial effects of phytochemical-rich foods on intestinal health, increasing attention has focused on combining dietary bioactive compounds with probiotic microorganisms [12]. Probiotics can interact with the intestinal environment, support epithelial homeostasis, and modulate inflammatory and oxidative responses, making them attractive ingredients for the development of functional foods [13]. Although probiotic research has traditionally focused on bacterial species, yeasts have recently emerged as promising alternatives owing to their intrinsic resistance to antibiotics, high tolerance to gastrointestinal conditions, and potential immunomodulatory properties [13]. These characteristics make probiotic yeasts attractive candidates for developing novel functional foods aimed at promoting intestinal health.

Among emerging probiotic yeasts, *Lachancea thermotolerans* has recently attracted interest because of its promising probiotic characteristics and suitability for functional food applications [14]. Agarbati et al. recently demonstrated that this species survives simulated gastrointestinal conditions, adheres to intestinal epithelial cells, and exerts antioxidant and immunomodulatory activities [15]. Moreover, this species has already been successfully incorporated into different fermented food matrices, highlighting its potential as an innovative ingredient for the development of functional foods [16,17]. Nevertheless, whether combining *L. thermotolerans* with a phytochemical-rich matrix, such as matcha, influences the profile of bioaccessible phytochemicals generated during digestion and enhances intestinal epithelial protection remains unknown.

Therefore, this study aimed to investigate the biological effects of digested matcha, *L. thermotolerans*, and their combination using an in vitro intestinal epithelial model. Specifically, we evaluated whether probiotic addition (i) modifies the antioxidant capacity and phytochemical profile of digested matcha, (ii) preserves intestinal barrier integrity under inflammatory conditions, and (iii) modulates the expression of inflammation-, antioxidant-, and barrier-related genes through a nutrigenomics approach.

## 2. Materials and Methods

### 2.1. Sample Preparations

#### 2.1.1. Digested Probiotic Yeast

The ability of a probiotic yeast to survive under the conditions of a simulated continuous digestion system, including competition with the gut microbiota, was assessed. It was also carried out in combination with matcha tea, a variety of green tea rich in antioxidant compounds.

In the present study, an emerging probiotic yeast, *L. thermotolerans* DiSVA322, was used. It is a native strain belonging to the Yeast Collection of the Department of Life and Environmental Sciences (DiSVA) of the Polytechnic University of Marche (Italy), previously isolated from grapes and characterized for its probiotic traits [15]. The yeast tested was maintained in YPD agar medium (10 g/L yeast extract, 20 g/L peptone, 20 g/L glucose and 18 g/L agar) at 4 °C for short-term storage and in YPD broth containing 40% (*w*/*v*) glycerol for long-term storage (−80 °C).

Before starting the digestion process, the yeast was cultured in 100 mL of YPD broth (20 g/L peptone, 10 g/L yeast extract, 20 g/L glucose) at 25 °C, 160 rpm for 24 h. The culture was centrifuged at 4000 rpm for 5 min; the supernatant was discarded, while the cell pellet was washed twice with sterile water. About 10^9^ viable cells were collected (concerning the probiotic claim) and suspended in a glass of “Levissima” mineral water (160 mL) with a fixed residue at 180 °C of 80 mg/L, sodium 2.1 mg/L, and pH 7.8, prior to digestion.

#### 2.1.2. Matcha Tea Preparation

**Figure 1:**
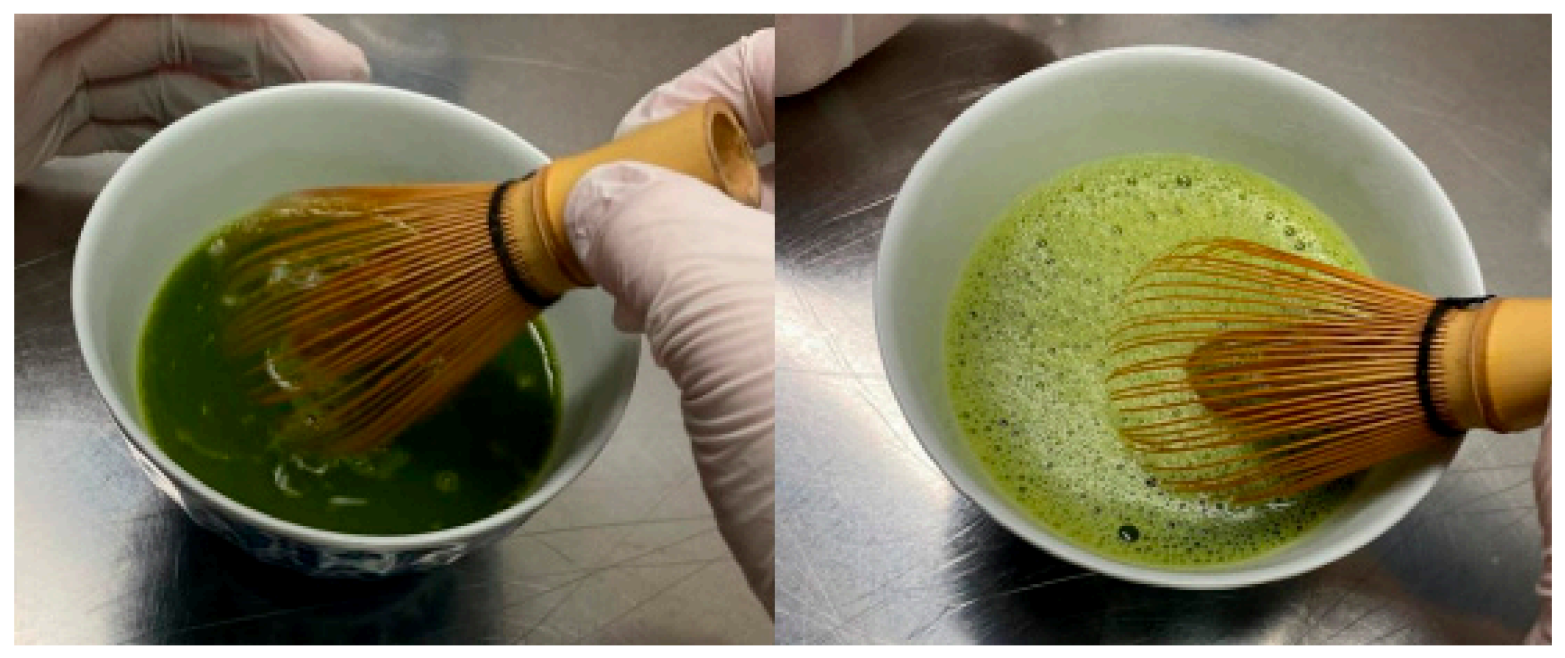
Matcha tea preparation.

Matcha tea “Starter” was kindly provided by “Cose di tè” Jesi, AN, Italy. Matcha (M) was prepared according to the company’s instructions. Briefly, 1.6 g of tea powder (Matcha Starter Kagoshima) was added to 160 mL of water at 70 °C in a ceramic teacup. Using a special bamboo whisk (chasen), zigzag movements were performed for two minutes, until the typical green foam formed on the surface (Figure 1). In the trial involving the simultaneous digestion of tea and yeast (M + P), approximately 10^9^ viable cells of DiSVA322 were added to the tea preparation, and the simulated digestive process was started.

### 2.2. In Vitro Digestion System

Matcha tea alone (M), the formulation containing the probiotic (M + P), and the probiotic alone (P) were subjected to in vitro gastrointestinal digestion as follows. A dynamic human gastrointestinal simulation system was configured using 2 L capacity bioreactors (Biostat, Sartorius, Göttingen, Germany) equipped with automated controls for temperature, pH, agitation, aeration, and fluid flows. The experimental setup was adapted from the Simulator of the Human Intestinal Microbial Ecosystem (SHIME) reactor feed framework [18,19] with minor modifications (Figure S1, Supplementary Material). The compositions of the simulated saliva, gastric juice, and intestinal juice were prepared according to the guidelines of the National Institute for Public Health and the Environment (RIVM) (Table S1, Supplementary Materials) [20]. The volumetric ratio of water, saliva, gastric juice, and intestinal medium was strictly maintained at 17:11:22:50 throughout the simulation process, respectively. The simulated digestion protocol initiated with an oral phase, where M, M + P, or P samples were mixed with the simulated saliva medium for 1 min at 37 °C under aerobic shaking conditions. The mixture was subsequently transferred to the gastric compartment containing the gastric medium for a 180 min incubation period (100 rpm, aerobic conditions). During this gastric phase, the pH was dynamically reduced from 5.0 to 3.0 over the first 120 min and further decreased to 2.0 during the final 60 min. Gastric emptying was simulated fractionally; five equal fractions were discharged every 30 min starting from the 30 min time point. Each emptied gastric fraction was progressively pumped into the intestinal vessel (duodenal phase) pre-filled with intestinal medium and incubated for 160 min (37 °C, 100 rpm, pH 6.0–6.5) under anaerobic conditions, which were maintained by a continuous N_2_ headspace flush.

This phase concluded 10 min after the introduction of the final gastric fraction and was immediately followed by a dialysis step designed to mimic physiological intestinal absorption and mitigate potential bile salt cytotoxicity. The chyme was continuously recirculated (20 mL/min) through a Diacap HiFlo high-flux dialyzer membrane (B. Braun, Melsungen, Germany) against a counter-current dialysis buffer (40 mL/min) containing 8.41 g/L NaCl and 1.13 g/L bovine serum albumin (pH 6.5). This procedure achieved a bile removal efficiency of ≥90% (wt/vol) within 70–80 min. Bile clearance was monitored spectrophotometrically by measuring the optical density at 350 nm against a linear calibration curve established with bile standards (0.5 to 5.0 g/L) diluted in the intestinal medium. Following dialysis, the colonic/ileal phase was simulated (4 h, 37 °C, pH 6.8–7.2, anaerobic environment) by inoculating the dialyzed medium with 20 mL of a freshly prepared human fecal microbiota suspension (10^6^ cells/mL).

### 2.3. L. thermotolerans DiSVA322 Viability

The ability of the probiotic yeast DiSVA322 to resist high-stress DYGIST conditions was assessed; at the end of the digestion process, viable cell counts were performed. Serial decimal dilutions were carried out in sterile water and plated on YPD agar medium. Plates were incubated at 25 °C prior to counting. This was done in triplicate for each trial. Results were expressed as Log viable cells, while the viability % was calculated considering the arithmetical data of inoculated cells (100% viability) and those that remained alive at the end of the system.

### 2.4. 2,2′-Azino-bis(3-ethylbenzothiazoline-6-sulphonic acid) (ABTS)

The antioxidant activity of the digested samples was determined using the 2,2′-azino-bis(3-ethylbenzothiazoline-6-sulphonic acid) (ABTS) assay. Briefly, the ABTS radical cation was produced by mixing a 7 mM ABTS stock solution with 2.45 mM potassium persulfate. The mixture was incubated in the dark for 16–18 h prior to use. The ABTS solution was then diluted to obtain an absorbance of 0.7–0.9 at 734 nm, measured using a FLUOstar Omega microplate reader (BMG LABTECH, Ortenberg, Germany).

Digested sample solutions at different concentrations were mixed with the ABTS working solution in a 96-well microplate, and the absorbance was measured at 734 nm using the FLUOstar Omega microplate reader (BMG LABTECH, Ortenberg, Germany). A blank was used for absorbance correction. Trolox solutions at different concentrations were used to generate a standard calibration curve, which was used to express the antioxidant capacity of the samples as Trolox equivalents. The experiment was performed in three replicates.

### 2.5. 2,2′-Diphenyl-1-picrylhydrazyl (DPPH)

The radical scavenging activity of the samples was evaluated using the 2,2′-diphenyl-1-picrylhydrazyl (DPPH) assay [21]. Briefly, a fresh prepared 200 µM DPPH solution was mixed with different concentrations of diluted digested samples in a 96-well microplate. The reaction mixtures were incubated in the dark at room temperature, and the absorbance was measured at 517 nm using the FLUOstar Omega microplate reader (BMG LABTECH, Ortenberg, Germany). A blank was used for absorbance correction. The antioxidant activity of digested samples was quantified using a Trolox calibration curve, and the results were expressed as Trolox equivalents (TE) of the digested products corrected for the dilution factor (1:20 *v*/*v*). The experiment was performed in three replicates.

### 2.6. Oxygen Radical Absorbance Capacity (ORAC)

The Oxygen Radical Absorbance Capacity (ORAC) assay was conducted as previously described [21]. In brief, fluorescein (0.08 μM in 75 mM phosphate buffer, pH 7.0) was used as the fluorescent probe, and reactions were carried out in black 96-well plates at 37 °C. Trolox (6.25–50 μM) was used to create the standard curve. Fluorescence was measured kinetically (excitation 485 nm, emission 530 nm) over 90 min, and results were expressed as TE calculated from the area under the curve (AUC), corrected for the dilution factor (1:20 *v*/*v*). The experiment was performed in triplicate.

### 2.7. Total Phenolic Content (TPC)

The total phenolic content (TPC) of the digested samples was determined using the Folin–Ciocalteu method [21]. Briefly, an aliquot of the diluted digested samples was mixed with Folin–Ciocalteu Reagent. After a short period of incubation, Sodium Carbonate solution was added to the reaction mixture. The samples were then incubated in the dark for 90 min, and the absorbance was measured at 725 nm using the FLUOstar Omega microplate reader (BMG LABTECH, Ortenberg, Germany). A blank was used for absorbance correction. Gallic acid was used as the standard for calibration and the results were expressed as gallic acid equivalents (GAEs) of digested products, corrected for the dilution factor (1:20 *v*/*v*). The experiment was performed in three replicates.

### 2.8. Caco-2 Cell Culture

Caco-2 cells, a human colonic epithelial cell line (ATCC, Rockville, MD, USA), were kindly provided by Professor Massimo Nabissi (University of Camerino). Caco-2 cells were cultured in Dulbecco’s modified Eagle’s medium (DMEM) supplemented with 10% heat-inactivated fetal bovine serum (FBS), 1% L-glutamine, 1% non-essential amino acids (NEAAs), and 1% penicillin/streptomycin. The cells were maintained at 37 °C in a humidified atmosphere with 5% CO_2_ and passaged when reaching 80% confluence.

### 2.9. Cell Viability Assay

The cytotoxic impact of the digested samples obtained from M, M + P, and P was assessed through the 3-(4,5-Di-2-yl)-2,5-ditetrazolium bromide (MTT) assay (Thiazolyl blue tetrazolium bromide 98%, code 158990050, Acros Organic, Fair Lawn, NJ, USA). In brief, Caco-2 cells were seeded in 96-well plates at a density of 1 × 10^4^ cells/well in complete medium and exposed to various concentrations of digested samples diluted in cell culture medium (1:2, 1:10, 1:20, 1:50, and 1:100 *v*/*v*) for 2 h. Following the incubation period, the cells were treated with a 5 mg/mL MTT solution. After 4 h, the absorbance was measured at 540 nm using a spectrometer reader (FLUOstar Omega, BMG LABTECH, Ortenberg, Germany). The experiment was performed in biological quadruplicates. The highest non-cytotoxic concentration (1:10 *v*/*v*) was selected for subsequent experiments. The same dilution factor was applied to all samples to ensure comparability across treatments as previously described in Bordoni et al. [22].

### 2.10. Treatments on Intestinal Monolayer

A Caco-2 monolayer cultured on Transwell inserts was used as an intestinal epithelial model to evaluate the effect of digested samples. Briefly, Caco-2 cells were seeded at a density of 26.23 × 10^3^ cells/cm^2^ onto Millicell 12-well hanging cell culture inserts (Millipore, cat: PCHT12H48, Sigma-Aldrich, St. Louise, MO, USA) placed in 12-well plates as previously described [21]. A volume of 0.5 mL of cell culture medium was added to the apical (AP) compartment, and 1.5 mL was added to the basolateral (BL) compartment. The medium was replaced every 2 days to prevent nutrient depletion. Cells were maintained at 37 °C in a humidified atmosphere with 5% CO_2_ and cultured until differentiation and complete epithelium formation.

Once the intestinal epithelial monolayer was fully established, as confirmed by TEER measurements, the digested samples obtained from M, M + P, and P were used to perform a 2 h pre-treatment on the monolayer.

Each pre-treatment was prepared by diluting each digested sample in cell culture medium at the highest non-cytotoxic concentration (1:10 *v*/*v*). Pre-treatments were carried out for 2 h in the AP compartment of the Transwell system to mimic human gastrointestinal residence, while the BL compartment contained only fresh cell culture medium. After incubation, the pre-treatment solutions in the AP compartment and the medium in the BL compartment were removed and replaced with fresh cell culture medium containing interleukin-1β (IL-1β) (SRP3083, Sigma–Aldrich, St. Louis, MO, USA) and Lipopolysaccharide (LPS) (Sigma–Aldrich, St. Louis, MO, USA). Briefly, LPS (10 μg/mL) was added to the AP compartment, while a combination of LPS (10 μg/mL) and IL-1β (10 ng/mL) was added to the BL compartment. The monolayers were incubated for 3 h to simulate short-term inflammation. A positive control (monolayers exposed to the inflammatory stimulus without pre-treatment, named “inflamed INF”) and a negative control (monolayers neither pre-treated nor exposed to the inflammatory stimulus, named “non-treated (NT)”) were included in the experiment. All the experiments were performed in biological triplicates. After the treatments, cells were collected, and cell pellets were frozen in liquid nitrogen and stored at −80 °C for subsequent analysis.

### 2.11. Intestinal Permeability (TEER)

The permeability of the Caco-2 monolayer was determined by measuring the trans-epithelial electrical resistance (TEER) values using a Millicell ERS (Electrical Resistance System) Voltohmmeter (Millipore, Burlington, MA, USA). Intestinal integrity was assessed during seeding to monitor the progression of intestinal epithelium formation. The resistance of the intestinal barrier was also measured at two distinct time points: after pre-treatments with digested samples and after inflammation induction. This was done to evaluate the potential protective effect of the digested samples against inflammation. Results are expressed as a percentage relative to the control (NT). Measurements were performed in triplicate for each well.

### 2.12. Gene Expression Analysis (RT-PCR)

Total RNA was purified from Caco-2 cells using the Total RNA Purification Plus Kit (Norgen Biotek, Thorold, ON, Canada) according to the manufacturer’s instructions and quantified using a NanoDrop ONE spectrophotometer (Thermo Fisher Scientific, Monza, Italy). Then, 1 μg of RNA was reverse transcribed to cDNA using the PrimeScript RT-PCR Kit (Takara Bio, Göteborg, Sweden), and quantitative real-time PCR (Biorad CFX96, Biorad laboratories, Hercules, CA, USA) was used to perform the gene expression analysis using TB Green Premix Ex TaqTM (Takara Bio, Göteborg, Sweden). The amplification conditions were: 30 s at 95 °C (denaturation), 5 s at 95 °C (annealing), and 30 s at 60 °C (extension), repeated for 40 cycles. The expression levels of the target genes were normalized relative to β-actin, using the 2–∆∆Ct method. Each analysis was run in technical duplicate. An inter-run calibrator sample was applied to adjust the results obtained from different amplification plates. Each PCR reaction was performed in technical duplicates. The target genes analyzed in Caco-2 monolayers were the tight junction genes (Zonulin, Occludin and Claudin); the pro-inflammatory genes (NF-κB, IL-8 and IL-1β); and the antioxidant-related genes (SOD1 and SOD2). Primer sequences used in the study are provided in Table S2 (Supplementary Materials).

### 2.13. Determination of Bioactive Compounds

#### 2.13.1. HPLC Analysis of 20 Polyphenolic Compounds

The determination of 20 polyphenolic compounds was carried out using a previously reported method with slight modifications [23]. Analyses were performed on a 1260 Infinity HPLC system (Agilent Technologies, Santa Clara, CA, USA) equipped with a diode array detector (DAD). Chromatographic separation was achieved using a Synergi Polar-RP C18 analytical column (4.6 mm × 250 mm, 4 µm; Phenomenex, Cheshire, UK). The mobile phase consisted of (A) water and (B) methanol, both containing 0.1% formic acid, delivered at a flow rate of 1 mL min^−1^ under gradient conditions as follows: 0 min, 20% B; 0–15 min, 20% B; and 15–45 min, 100% B. The column temperature was maintained at 30 °C, and the injection volume was 10 µL. UV spectra were acquired between 210 and 400 nm for all compounds. Quantification wavelengths were selected according to the maximum absorbance of each analyte: 210 nm for ursolic and oleanolic acids; 230 nm for procyanidin A2 and procyanidin B2; 265 nm for rutin, quercetin-3-D-galactoside, and kaempferol-3-glucoside; 272 nm for gallic acid; 280 nm for (+)-catechin hydrate, (−)-epicatechin, phloretin, and phlorizin; 325 nm for chlorogenic acid, neochlorogenic acid, caffeic acid, p-coumaric acid, and trans-ferulic acid; 520 nm for cyanidin-3-glucoside; and 365 nm for kaempferol and quercetin. Quantification of all compounds was performed using calibration curves constructed from injections of the corresponding analytical standards.

#### 2.13.2. Caffeine, Chlorogenic and Phenolic Acids Analysis

The quantification was carried out using a previously reported method [24] with slight modifications. The analysis was performed using an Agilent 1100 HPLC system equipped with a diode array detector (DAD), a binary pump, and an autosampler. The column temperature was maintained at 40 °C. Chromatographic separation was achieved using a Gemini C18 analytical column (250 mm × 3.0 mm, 5 µm; Phenomenex), protected by a Security Guard C18 pre-column (4 cm × 3 mm, 5 µm; Phenomenex). Elution was performed under gradient conditions using water (A) and methanol (B), both acidified with 0.1% formic acid, as the mobile phases. The gradient program was set as follows: 0–10 min, isocratic at 20% B; 10–15 min, increased to 35% B; 15–20 min, increased to 55% B; at 20 min, isocratic at 85% B, followed by a return to 20% B within 5 min. The flow rate was maintained at 0.8 mL min^−1^. A volume of 3 μL was injected for each analysis. Detection wavelengths were selected according to the analytes: 325 nm for chlorogenic acids, 270 nm for caffeine, 310 nm for p-coumaric acid, and 280 nm for trans-cinnamic acid.

### 2.14. Statistical Analysis

Statistical analysis was performed using SPSS Version 30 (IBM SPSS Statistics for Mac, Armonk, NY, USA), and JMP Pro, version 18.0.2 (SAS Institute Inc., Cary, NC, USA). Differences between group means were assessed using one-way ANOVA followed by Tukey’s post hoc test for multiple comparisons or the Kruskal–Wallis test. Also, independent *t*-tests were used for comparisons between two groups, when appropriate. A *p*-value < 0.05 was considered statistically significant throughout the study. Results are shown as means ± SD.

## 3. Results

### 3.1. Antioxidant Capacity of the Digests (ABTS, ORAC, DPPH)

**Figure 2:**
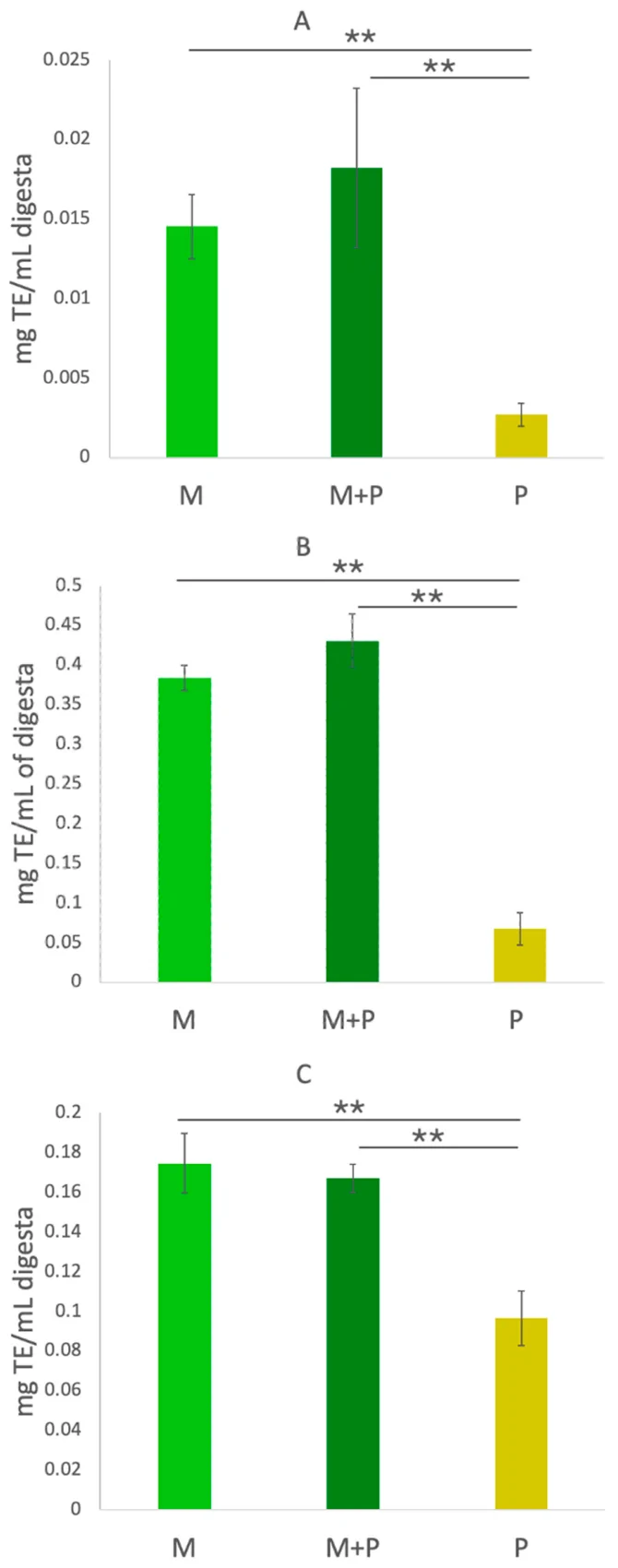
(**A**) DPPH, (**B**) ABTS, and (**C**) ORAC assays after in vitro digestion of matcha tea (M); matcha tea added with the probiotic (M + P); and the probiotic alone (P), expressed as mg TE/mL of digested product. Data are represented as the mean ± SD of three replicates. ** *p* < 0.01.

The antioxidant activity of digested samples obtained from M, M + P, and P was assessed using ORAC, ABTS, and DPPH assays. Figure 2 shows that the digested M (*p* < 0.01 vs. P) and M + P (*p* < 0.01 vs. P) presented a higher antioxidant capacity than P (overall *p* < 0.01). No significant difference was measured when comparing M and M + P.

### 3.2. Total Polyphenol Content (TPC) of Digested Samples

**Figure 3:**
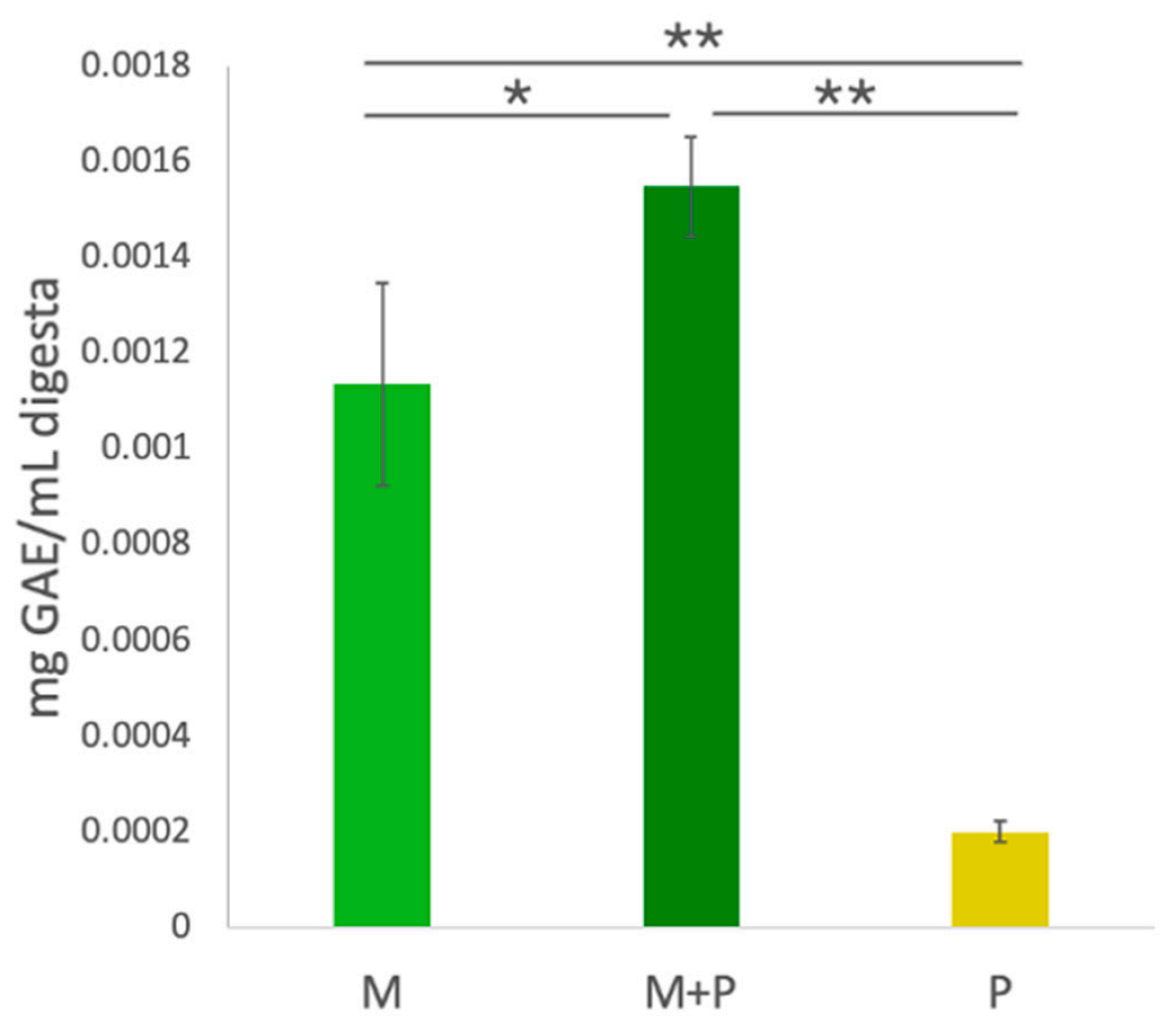
Total polyphenol content of M, M + P, and P expressed as mg GAE/mL of digested product. Data are represented as the mean ± SD of three replicates. M = digested matcha tea; M + P = digested matcha tea added with the probiotic; P = digested probiotic alone. * *p* < 0.05; ** *p* < 0.01.

The total polyphenol content (TPC) of the digested samples was expressed as gallic acid equivalents [(mg GAE)/mL] of digested products. Figure 3 shows that the digested M (*p* < 0.01; M vs. P) and M + P (*p* < 0.01; M + P vs. P) showed a higher total polyphenol content than P (overall *p* < 0.01). Furthermore, M + P revealed a polyphenol content higher than M (*p* < 0.05; M + P vs. M).

### 3.3. Cell Viability Results

**Figure 4:**
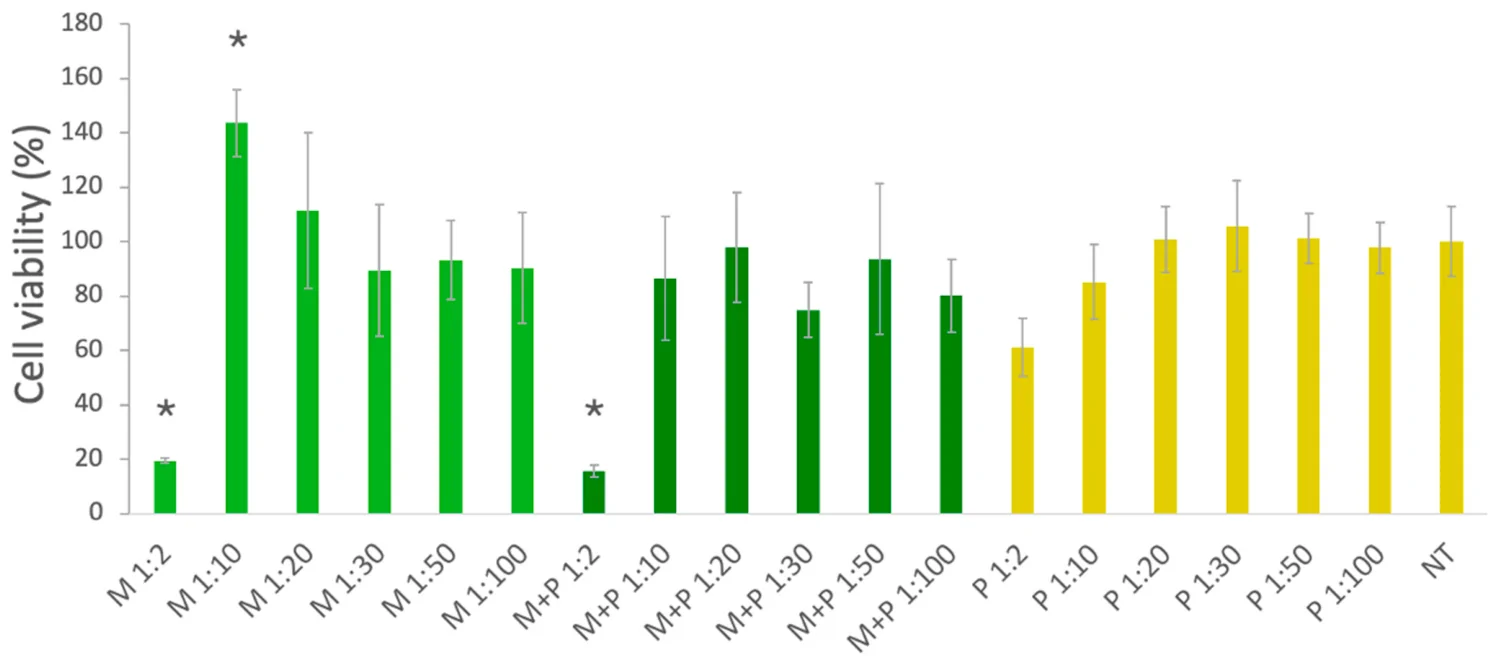
Assessment of cytotoxicity of the digested samples (M, M + P, and P) on Caco-2 after 2 h exposure to different digested sample concentrations (from 1:2 to 1:100 *v*/*v* prepared in DMEM). M = digested matcha tea; M + P = digested matcha tea added with the probiotic; P = digested probiotic alone; NT = untreated cells. * *p* < 0.05; vs. NT).

Figure 4 shows the cell viability of Caco-2 cells after 2 h treatment with the digests (M, M + P, and P). The MTT assay revealed that exposure to M and M + P at a 1:2 (*v*/*v*) dilution was cytotoxic and significantly different compared to untreated cells (NT). Specifically, they showed a decrease in viability of 80.7 ± 0.8% and 84.5 ± 2.0%, respectively. (*p* < 0.05, M 1:2 vs. NT; *p* < 0.05 M + P 1:2 vs. NT; Overall *p* < 0.05). Furthermore, the cells treated with M 1:10 showed a significant difference in viability compared to NT cells (*p* < 0.05).

### 3.4. Permeability Assay Results

The integrity of the Caco-2 monolayers, used as an intestinal epithelium model, was evaluated by measuring the TEER values at two time points: after the pre-treatment with digested products (T 2 h) and after the exposure of the monolayer to the inflammatory stimulus (T 3 h).

According to the results, no difference between groups in TEER values was measured after the 2 h pre-treatment.

**Figure 5:**
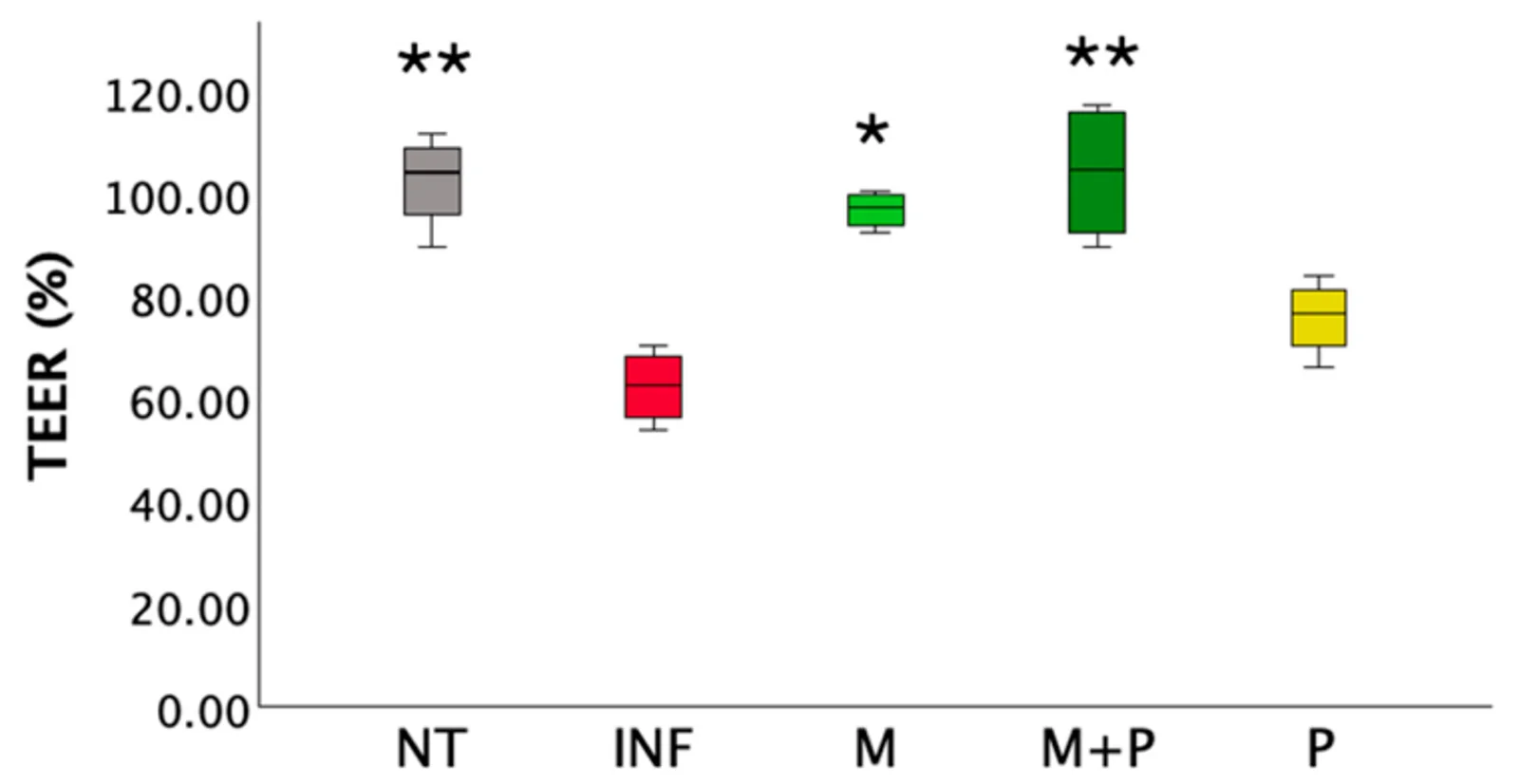
Effect of the digested products on intestinal monolayer integrity. TEER values were measured in Caco-2 monolayers pre-treated for 2 h with digested products and then exposed to inflammation for 3 h. (NT = untreated Caco-2 monolayers and INF = cells exposed only to the inflammatory stimulus). Results are expressed as % of untreated control (NT). M = digested matcha tea; M + P = digested matcha tea added with the probiotic; P = digested probiotic alone; NT = non-treated control; INF = inflamed control. * *p* < 0.05, ** *p* < 0.01, vs. inflamed cells (INF).

A significant difference between groups (overall *p* = 0.01) was measured after the 3 h short-term inflammation induced on the monolayers. In particular, the integrity of the Caco-2 monolayer was significantly different when comparing untreated cells (NT) and cells exposed only to the inflammatory stimulus (INF), with significantly lower TEER values observed in the inflamed group (INF) (*p* < 0.01), indicating that inflammation was successfully induced. Furthermore, cells pre-treated with M (*p* < 0.05) and M + P (*p* < 0.01) and subsequently exposed to the inflammatory stimulus exhibited significantly higher TEER values compared to cells exposed only to inflammation (Figure 5).

### 3.5. Expression Levels of Tight Junction Genes

**Figure 6:**
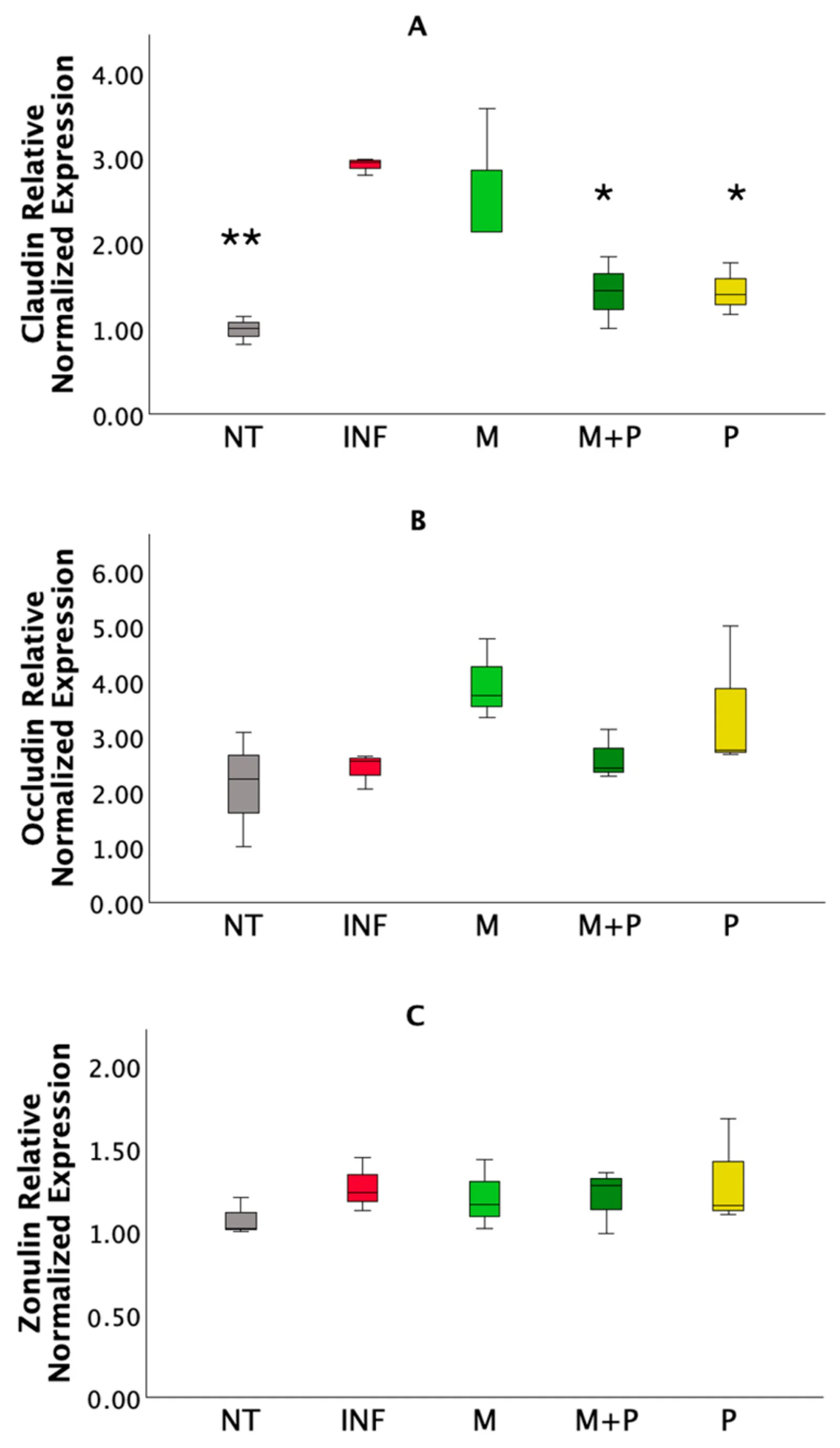
Gene expression levels of tight junctions measured by RT-PCR. Expression levels of Claudin (**A**), Occludin (**B**), and Zonulin (**C**) in untreated Caco-2 monolayer (NT), in monolayers exposed to 2 h pre-treatments with digested products (M, M + P, P) followed by a 3 h short-term inflammatory stimulus, and in monolayers exposed to the inflammatory stimulus alone (INF). M = digested matcha tea; M + P = digested matcha tea added with the probiotic; P = digested probiotic alone; NT = non-treated control; INF = inflamed control. * *p* < 0.05, ** *p* < 0.01 vs. INF.

The gene expression levels of the tight junction genes (Claudin, Occludin and Zonulin) were measured in the untreated Caco-2 monolayers (NT); in monolayers exposed to 2 h pre-treatments with digested products followed by a 3 h short-term inflammatory stimulus; and in monolayers exposed to the inflammatory stimulus alone (INF), as shown in Figure 6. A statistically significant increase in the expression level of the Claudin gene was measured in INF compared to NT (*p* < 0.01). Furthermore, a statistically significant decrease in the Claudin gene expression level was detected in cells pre-treated with M + P (*p* = 0.016) and P (*p* = 0.018) and then inflamed compared to INF (overall *p* < 0.01). No significant difference between groups was measured in Occludin and Zonulin gene expression levels.

### 3.6. Expression Levels of Pro-Inflammatory Genes

**Figure 7:**
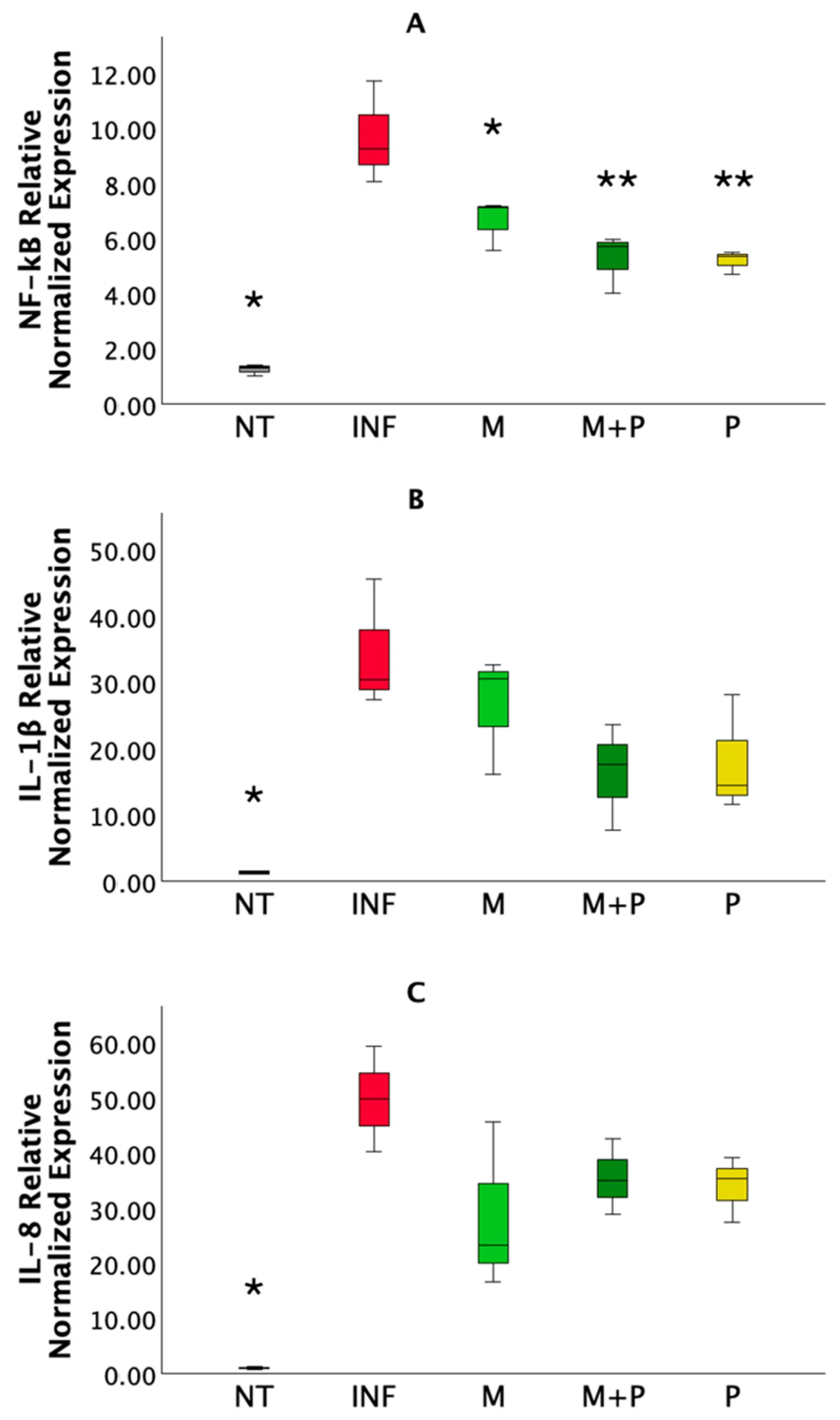
Expression levels of inflammatory genes measured by RT-PCR. Expression levels of NF-κB (**A**), IL-8 (**B**), and IL-1β (**C**) in untreated Caco-2 monolayer (NT); in monolayers exposed to 2 h pre-treatments with digested products (M, M + P, P) followed by a 3 h short-term inflammatory stimulus; and in monolayers exposed to the inflammatory stimulus alone (INF). M = digested matcha tea; M + P = digested matcha tea added with the probiotic; P = digested probiotic alone; NT = non-treated control; INF = inflamed control. * *p* < 0.05; ** *p* < 0.01 vs. INF.

The gene expression levels of the pro-inflammatory and anti-inflammatory genes (NF-κB, IL-8 and IL-1β) were measured in the untreated Caco-2 monolayers (NT), in monolayers exposed to 2 h pre-treatments with digested products followed by a 3 h short-term inflammatory stimulus, and in monolayers exposed to the inflammatory stimulus alone (INF) (Figure 7). A statistically significant increase in the expression level of the NF-κB gene was measured in the INF sample compared to NT (*p* < 0.01). A statistically significant decrease in the NF-κB gene expression level was detected in the cells pre-treated with M + P (*p* = 0.008) and P (*p* = 0.007) and then with inflamed INF compared to INF (overall *p* < 0.001).

A statistically significant increase in the expression level of IL-8 and IL-1β genes was measured in the INF group compared to NT (*p* < 0.05; overall *p* < 0.01), indicating that inflammation was successfully induced.

### 3.7. Expression Levels of Antioxidant Genes

**Figure 8:**
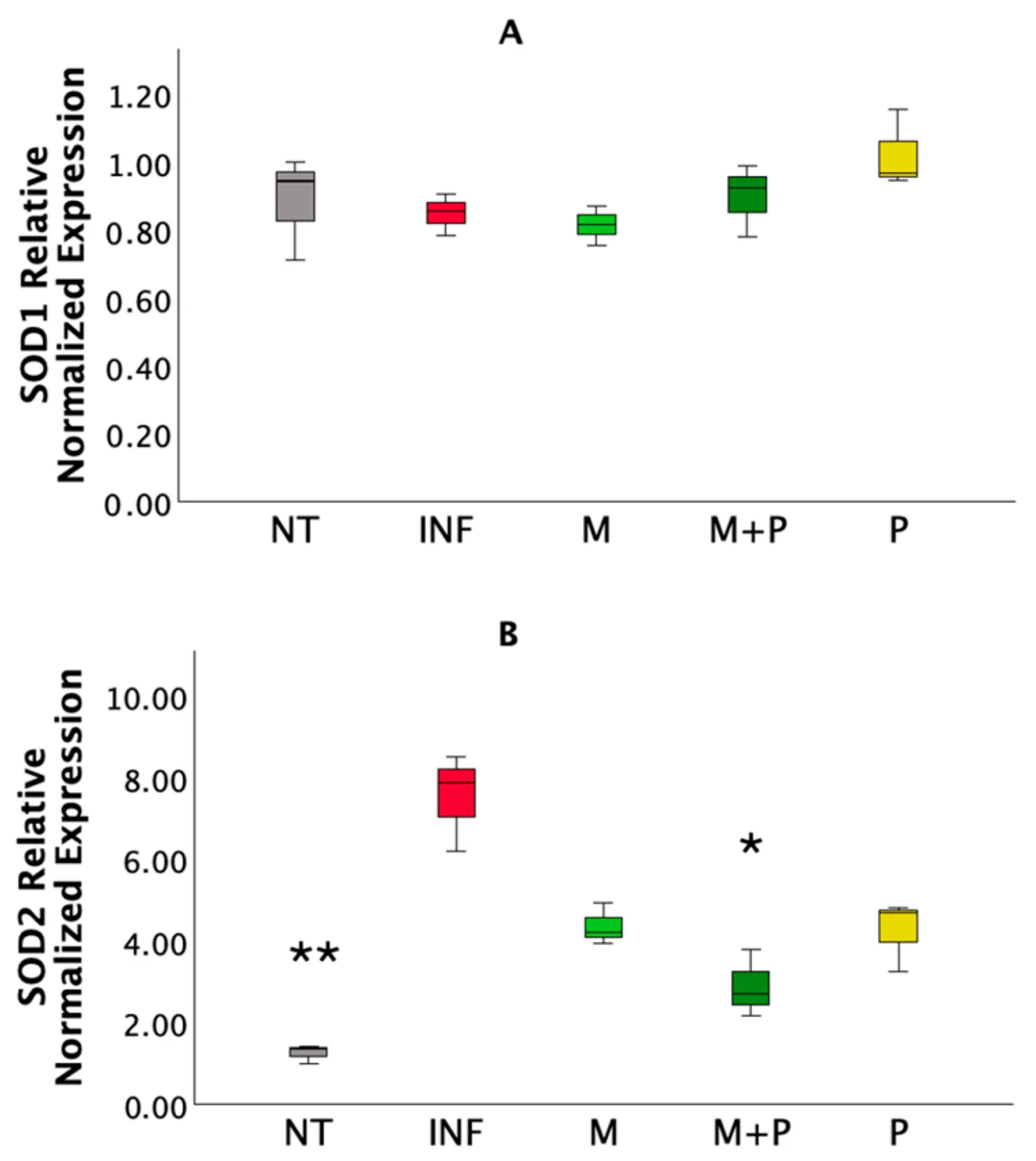
Expression levels of antioxidant genes measured by RT-PCR. Expression levels of SOD1 (**A**) and SOD2 (**B**) in untreated Caco-2 monolayer (NT); in monolayers exposed to 2 h pre-treatments with digested products (M, M + P, P) followed by a 3 h short-term inflammatory stimulus; and in monolayers exposed to the inflammatory stimulus alone (INF). M = digested matcha tea; M + P = digested matcha tea added with the probiotic; P = digested probiotic alone; NT = non-treated control; INF = inflamed control. * *p* < 0.05, ** *p* < 0.01 vs. INF.

Figure 8 shows the gene expression levels of genes involved in the antioxidant response (SOD1 and SOD2) in Caco-2 cells after 2 h of treatment with the digests and 3 h of induced inflammation with LPS and IL-1B. A statistically significant increase in the expression level of the SOD2 gene was measured in INF compared to NT (*p* < 0.01). Furthermore, a statistically significant decrease in SOD2 gene expression level was detected in the cells pre-treated with M + P (*p* < 0.05) and then inflamed compared to INF (overall *p* = 0.003). No significant difference between groups was measured in SOD1 gene expression level.

### 3.8. Viability of Probiotic Yeast

The survival capability of the probiotic yeast strain *L. thermotolerans* DiSVA322 within the challenging environment of the human gastrointestinal tract was assessed using two distinct delivery vehicles: mineral water and matcha tea. Viability data corresponding to each vehicle are detailed in Table 1. The probiotic yeast demonstrated robust resistance to the stressful conditions of the DYGIST system regardless of the matrix used. At the end of the simulated digestion, the viable cell counts reached 7.81 ± 0.03 Log CFU/mL for mineral water and 8.93 ± 0.10 Log CFU/mL for matcha tea, compared to initial inoculation levels of approximately 8.64 and 9.38 Log CFU/mL, respectively.

These results correspond to an absolute survival rate of 14.95 ± 0.03% when administered in water, and a significantly higher rate of 35.15 ± 0.10% when delivered within the matcha tea matrix, assuming 100% viability at baseline. Crucially, despite the observed reduction in overall cell counts, the final concentration remained well within the logarithmic threshold required to satisfy official probiotic claims.

### 3.9. HPLC Profile and Quantification of Polyphenolic Compounds 

Table 2 reports the variation in the content of approximately twenty of the most common phenolic compounds in each in vitro digested sample, as determined by HPLC-DAD. No polyphenols were detected in the digested sample obtained from P. In contrast, eight polyphenols were identified in the digested fraction M. Among these, rutin was the most abundant (6.68 ± 0.38 mg L^−1^), followed by gallic acid (3.16 ± 0.02 mg L^−1^) and epicatechin (2.08 ± 0.43 mg L^−1^). In M + P, the concentrations of gallic acid (13.35 ± 0.01 mg L^−1^) and epicatechin (3.07 ± 0.03 mg L^−1^) were significantly higher than those measured in matcha tea alone (*p* < 0.05).

### 3.10. Caffeine, Chlorogenic and Phenolic Acid Profile 

Regarding chlorogenic acid compounds, 3-caffeoylquinic acid (3-CQA) was identified as the most abundant compound in digested M and M + P, followed by 3,5-caffeoylquinic acid (3,5-CQA). Caffeine was also detected in the same fraction at relatively high concentrations. No chlorogenic acids were detected in the digested sample obtained from P. The data presented in Table 3 show a significant reduction in caffeine and 3,5-CQA concentrations in digested M + P compared to M, while no statistically significant difference was observed for 3-CQA.

## 4. Discussion

In this study, we demonstrated that matcha tea (M) formulated with the probiotic yeast *L. thermotolerans* DiSVA322 (P) modulated the phytochemical profile generated after gastrointestinal digestion while preserving the antioxidant capacity of the digested product. More importantly, the digested formulation protected intestinal epithelial integrity under inflammatory conditions and modulated the expression of genes involved in inflammation and antioxidant defense.

Considering the antioxidant activity, no significant differences were detected between M and M + P after digestion, suggesting that the combination M + P maintained a comparable antioxidant potential to M alone under gastrointestinal conditions and demonstrating that P did not alter matcha antioxidant capacity. Interestingly, after in vitro digestion, M + P exhibited a higher total polyphenol content, measured by TPC, compared to M alone.

To validate this result, HPLC analysis was performed on our digested samples: eight polyphenols were identified in both M and M + P digests (procyanidin B2, rutin, kaempferol-3-glucoside, gallic acid, epicatechin, ρ-coumaric acid, trans-ferulic acid and quercetin).

Among these, rutin was the most abundant compound in M, followed by gallic acid and epicatechin. These results are consistent with those reported by Gómez-Mejía and Rusak et al., who observed, respectively, similar trends for gastric- and duodenal-digested green tea and the absence of catechin in the intestinal phase [25,26].

Considering M + P, gallic acid and epicatechin were the most abundant phenolic compounds, with concentrations higher than those measured in M. In particular, the concentration of gallic acid was approximately 322% higher in M + P digests compared to M digesta. Since both samples underwent the same gastrointestinal digestion protocol, this difference suggests that the presence of *L. thermotolerans* may have influenced the release, transformation, or stability of phenolic compounds during digestion, thereby modulating the phytochemical composition of the resulting digesta. On the other hand, it was demonstrated that the probiotic yeast *Saccharomyces cerevisiae* enhances the bioaccessibility of phenolic compounds by serving as a protective delivery system throughout in vitro digestion [27,28]. Given that caffeine is well known to be present in matcha tea [29], we used HPLC analysis to further investigate the chlorogenic acid content of our samples.

Results revealed that 3-caffeoylquinic acid (3-CQA), 3,5-caffeoylquinic acid (3,5-CQA) and caffeine were present in both M and M + P. However, the content of caffeine and 3,5-CQA was lower in M + P than in M. In particular, the caffeine content decreased by approximately 40.5% in M + P compared to M. Since M and M + P underwent the same digestion procedure, the lower caffeine content observed in M + P may be associated with the presence of *L. thermotolerans* DiSVA322. Although this finding suggests that the yeast may contribute to the reduction in caffeine content, the underlying mechanism cannot be conclusively established from the present data. The observed reduction may result from yeast-mediated degradation of caffeine, but alternative explanations, such as the adsorption or association of caffeine with the yeast biomass, as well as changes in its distribution or partitioning during the dialysis process, cannot be excluded. Therefore, the reduction in caffeine content observed in M + P should be interpreted as an effect associated with the presence of the yeast rather than as direct evidence of caffeine metabolism. Nevertheless, the marked difference in caffeine content between M and M + P highlights the potential role of *L. thermotolerans* DiSVA322 in modifying the chemical composition of the digested matrix.

In recent years, microbial fermentation has been widely used to reduce the caffeine content of coffee. However, little is known about the use of non-*Saccharomyces* yeasts for this purpose, although several new caffeine-degrading yeast strains have been identified [30]. Liu et al. reported that inoculation of *L. thermotolerans* into spent coffee grounds did not result in any significant changes in caffeine content [31]. The discrepancy between their findings and our results may be related to differences in the food matrix and experimental conditions, which may affect the interaction between caffeine and the microbial biomass and/or its fate during processing. Concerning the biological activity of the digesta, M and M + P at a 1:2 (*v*/*v*) concentration showed cytotoxicity toward Caco-2 cells. This effect may be related, at least in part, to the high polyphenol content of these extracts, which may contribute to cellular stress and loss of viability at high concentrations.

M and M + P, at the highest non-cytotoxic concentration tested, exerted a protective effect against inflammation-induced epithelial damage, as shown by TEER measurements. This beneficial effect may be attributed to the presence of bioactive compounds in both samples. Indeed, polyphenols have been suggested to regulate intestinal permeability and inflammatory/antioxidant pathways [32,33]. The higher content of polyphenols in M + P compared to M may be hypothesized to contribute to its molecular effect: M + P, but not M, was able to counteract the inflammation-induced increase in Claudin gene expression. We speculate that the upregulation of the Claudin gene observed in inflamed cells compared to untreated cells may represent a c-adaptative molecular response to inflammatory stress aimed at counteracting inflammation-induced alterations in gene expression. Interestingly, M + P prevented the increase in Claudin gene expression, suggesting that M + P may modulate the molecular response of the epithelium to inflammatory stress. These findings indicate that the formulation of matcha added with *L. thermotolerans* not only preserved barrier functionality, as demonstrated by TEER measurements, but also induced a molecular signaling cascade by modulating Claudin gene expression. A similar molecular effect was exerted also by P; however, no corresponding improvement in TEER values was detected, suggesting that P activated intracellular molecular signals without producing an immediate functional effect.

Concerning the regulation of inflammatory genes, all three digests downregulated NF-κB gene expression, supporting an anti-inflammatory nutrigenomic response. However, although a consistent downward trend was observed for both IL-1β and IL-8 in treated cells, the differences did not reach statistical significance. This finding reflects the complexity of inflammatory signaling, in which cytokine expression is regulated by multiple interacting pathways beyond NF-κB alone [21,34,35,36]. Thus, the observed effects suggest a potential modulation of the inflammatory response, supported by the significant downregulation of NF-κB, while the lack of statistically significant changes in IL-1β and IL-8 does not allow us to conclude that the formulation exerts an anti-inflammatory effect.

On the contrary, only M + P downregulated the inflammation-induced increase in SOD2 gene expression, while SOD1 was not regulated by any treatment. This result suggests a compensatory mechanism against inflammation-induced stress specifically at the mitochondrial level.

## 5. Conclusions

Taken together, these findings suggest that probiotic enrichment not only preserves but enhances the nutrigenomic effect of matcha tea. Concurrently, the co-ingestion of the probiotic yeast with matcha tea promotes yeast survival compared to its administration in plain mineral water, likely facilitating the expression of its health-promoting probiotic functionalities. The increase in polyphenol content, together with the decrease in caffeine content, indicates a modulation of the bioactive profile that may contribute to an improved nutrigenomic effect of the formulation. Furthermore, the presence of probiotics does not impair the protective effects of matcha on intestinal permeability and, instead, appears to reinforce its activity by modulating molecular signals involved in tight junction regulation.

These results suggest that the probiotic-enriched matcha formulation could be used in the development of food products enriched with antioxidant bioactive compounds, with potential beneficial effects on intestinal health.

This study has limitations that should be addressed. The experiments were conducted in vitro, lacking the complexity of the in vivo intestinal environment. The translational relevance of these findings to physiological conditions remains to be further studied in vivo. Furthermore, the investigation of the molecular mechanisms in this study was based on gene expression levels, without measuring protein levels, enzymatic activities, or pathway activation. Although changes in mRNA expression may provide valuable insights into the biological processes investigated, they do not necessarily translate into corresponding changes at the protein level or directly reflect functional alterations. Therefore, the absence of protein-level and functional validation represents a limitation of the present study. Nevertheless, the aim of this study was to identify preliminary molecular trends that may provide insights into the potential mechanisms of action. Future studies incorporating complementary approaches, such as Western blotting, ELISA, immunofluorescence, enzymatic assays, or functional assays, will be important to confirm and further characterize these findings at the protein and functional levels. Thus, this work should be considered a preliminary investigation that generates hypotheses for future studies aimed at validating and extending the present findings. Despite this limitation, the present study provides initial evidence of potential translational relevance. Matcha tea is already widely consumed and naturally rich in bioactive compounds; thus, its enrichment with *L. thermotolerans* could represent an easy strategy to further enhance its properties. In this context, the observed beneficial effects on intestinal permeability, together with the improved chemical profile of the enriched matcha tea, highlight its promising role as a functional food targeting intestinal health.

## Figures and Tables

**Table 1:** Viability of the probiotic yeast DiSVA322 after the digestion process. The viability % refers to data expressed in arithmetical mode (no Log expression), and it was calculated based on the cells that remained alive at the end of digestion (End DYGIST), considering the 100% viability of inoculated cells (Start DYGIST). Data are reported as mean value ± standard deviation of three replicates.

Trials	Start DYGIST	End DYGIST	
	Log Viable Cells	Log Viable Cells	Viability %
DiSVA322 + mineral water	8.64 ± 0.05	7.81 ± 0.03	14.95 ± 0.03
DiSVA322 + matcha tea	9.38 ± 0.15	8.93 ± 0.10	35.15 ± 0.10

**Table 2:** Phenolic compound profile for samples after in vitro digestion. Data are reported as mg L^−1^ of digested extract and expressed as mean ± standard deviation. Mean values marked with different letters within the same column denote statistically significant differences. M = digested matcha tea; M + P = digested matcha tea added with the probiotic; P = digested probiotic alone; n.d. = not detected.

Compound	P	M	M + P
Ursolic acid	n.d.	n.d.	n.d.
Oleanolic acid	n.d.	n.d.	n.d.
Procyanidin A2	n.d.	n.d.	n.d.
Procyanidin B2	n.d.	0.57 ± 0.34 ^a^	0.94 ± 0.26 ^a^
Rutin	n.d.	6.68 ± 0.38 ^a^	5.17 ± 1.3 ^b^
Quercetin-3-D-galactoside	n.d.	n.d.	n.d.
Kaempferol-3-glucoside	n.d.	0.59 ± 0.01 ^a^	0.62 ± 0.05 ^a^
Gallic acid	n.d.	3.16 ± 0.02 ^b^	13.35 ± 0.01 ^a^
Catechin	n.d.	n.d.	n.d.
Epicatechin	n.d.	2.08 ± 0.43 ^b^	3.07 ± 0.03 ^a^
Phloretin	n.d.	n.d.	n.d.
Phlorizin	n.d.	n.d.	n.d.
Chlorogenic acid	n.d.	n.d.	n.d.
Neochlorogenic acid	n.d.	n.d.	n.d.
Caffeic acid	n.d.	n.d.	n.d.
ρ-Coumaric acid	n.d.	0.36 ± 0.07 ^a^	0.41 ± 0.06 ^a^
Trans-ferulic acid	n.d.	0.48 ± 0.14 ^b^	0.65 ± 0.02 ^a^
Cyanidin-3-glucoside	n.d.	n.d.	n.d.
Kaempferol	n.d.	n.d.	n.d.
Quercetin	n.d.	0.33 ± 0.10 ^a^	0.34 ± 0.10 ^a^

**Table 3:** Caffeine, chlorogenic and phenolic acids profiles for samples after in vitro digestion. Data are reported as mg L^−1^ of digested extract and expressed as mean ± standard deviation. Mean values marked with different letters within the same column denote statistically significant differences. M = digested matcha tea; M + P = digested matcha tea added with the probiotic; P = digested probiotic alone; n.d. = not detected.

Compound	P	M	M + P
3-CQA	n.d.	9.82 ± 1.11 ^a^	8.96 ± 2.12 ^a^
caffeine	n.d.	84.83 ± 0.41 ^a^	50.73 ± 0.46 ^b^
5-CQA	n.d.	n.d.	n.d.
4-CQA	n.d.	n.d.	n.d.
p-cumaric	n.d.	n.d.	n.d.
3,5-CQA	n.d.	5.10 ± 0.42 ^a^	3.87 ± 0.00 ^b^
trans-cinnamic	n.d.	n.d.	n.d.

## Data Availability

The authors confirm that the data supporting the findings of this study are available within this article and its Supplementary Materials.

## Supplementary Materials

The following supporting information can be downloaded at https://www.mdpi.com/article/10.3390/nu18183004/s1. Figure S1. Schematic representation of the digestive system: mouth phase, gastric phase, and intestinal phase. Table S1. Composition of the medium that represents saliva, gastric and intestinal juices. Table S2. Primer sequences for housekeeping genes and genes of interest for qPCR.

## Abbreviations

The following abbreviations are used in this manuscript

ABTS	2,2′-Azino-bis(3-ethylbenzothiazoline-6-sulfonic acid)
AP	Apical Compartment
AUC	Area Under the Curve
BL	Basolateral Compartment
BSA	Bovine Serum Albumin
Caco-2	Human Colorectal Adenocarcinoma Cell Line
cDNA	Complementary DNA
CO_2_	Carbon Dioxide
DAD	Diode Array Detector
DMEM	Dulbecco’s Modified Eagle Medium
DPPH	2,2′-Diphenyl-1-picrylhydrazyl
DYGIST	Dynamic Gastrointestinal Simulator
EGCG	Epigallocatechin Gallate
FBS	Fetal Bovine Serum
GAEs	Gallic Acid Equivalents
HPLC	High-Performance Liquid Chromatography
IL-1β	Interleukin-1 Beta
IL-8	Interleukin-8
INF	Inflamed Control
LPS	Lipopolysaccharide
M	Matcha Tea
M + P	Matcha Tea Supplemented with Probiotic Yeast
MTT	3-(4,5-Dimethylthiazol-2-yl)-2,5-diphenyltetrazolium bromide
NEAAs	Non-Essential Amino Acids
NF-κB	Nuclear Factor kappa B
NT	Non-Treated Control
ORAC	Oxygen Radical Absorbance Capacity
P	Probiotic Yeast
PCR	Polymerase Chain Reaction
qPCR	Quantitative Polymerase Chain Reaction
RNA	Ribonucleic Acid
RT-PCR	Reverse Transcription Polymerase Chain Reaction
SHIME	Simulator of the Human Intestinal Microbial Ecosystem
SOD1	Superoxide Dismutase 1
SOD2	Superoxide Dismutase 2
TEs	Trolox Equivalents
TEER	Trans-epithelial Electrical Resistance
TPC	Total Phenolic Content
YPD	Yeast Extract Peptone Dextrose (Medium)
ZO-1	Zonula Occludens-1
