# Supplementary material for: A Matcha Tea Formulation Containing Lachancea thermotolerans: Phytochemical Profile After In Vitro Digestion and Effects on Intestinal Epithelial Function
Source: Nutrients. 2026 Sep 14;18(18):3004. doi: 10.3390/nu18183004 (PMC13610030; doi:10.3390/nu18183004)
Supplement: Supplementary file 1 [file nutrients-18-03004-s001.zip › nutrients-4470352-supplementary.pdf]

Figure S1. Schematic representation of the digestion system: mouth phase, gastric phase, and intestinal phase.

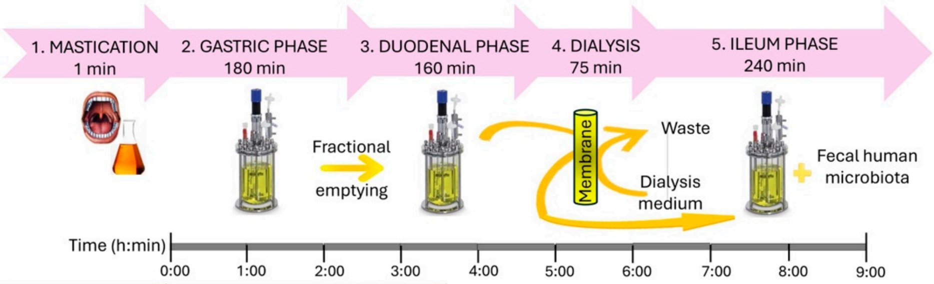

Time (min)	1'	0'- 30'	60'	90'	120'	150'	180'
Anatomical district	MOUTH	STOMACH					
Volum (mL)	166 (H_2O/meal) + 112 (Saliva)	500	400	300	200	100	0
Conditions	37 °C - 100 rpm	37 °C - 100 rpm - Aerobic					
Sampling	*						

30'	60'	90'	120'	160'	75'	240'
DUODENUM					DIALYSIS	ILEUM
600	700	800	900	1000	from 1000-to 250	250 + 20 mL fecal human microbiota
37 °C - 100 rpm – Anaerobic					Aerobic	37 °C - 100 rpm – Anaerobic
						*

Table S1. Composition of the medium that represents saliva, gastric and intestinal juices.

Compounds	Saliva medium (g/L)	Gastric medium (g/L)	Intestinal medium (g/L)
NaCl	0.30	2.75	6.43
$NaH_2PO_4 \cdot 2H_2O$	1.15	0.35	
KCl	0.90	0.82	0.50
NH_4Cl	0.31		
KSCN	0.20		
$Na_2SO_4 \cdot 10H_2O$	1.29		
$CaCl_2 \cdot 2H_2O$		0.40	0.21
KH_2PO_4			0.05
$MgCl_2 \cdot 6H_2O$			0.03
$NaHCO_3$			5.67
Mucin from porcine stomach type II (Sigma-Aldrich, St. Louis, Missouri, USA)	0.05	3.00	

Urea	0.20	0.09	0.15
Uric acid	0.02		
D-Glucuronic acid		0.02	
D-(+)-Glucosamine hydrochloride		0.33	
Bovine serum albumin (Sigma-Aldrich, St. Louis, Missouri, USA)		2.25	1.27
Pepsin from porcine stomach mucosa (Sigma-Aldrich, St. Louis, Missouri, USA)		4.50	
Pancreatin from porcine pancreas (Sigma-Aldrich, St. Louis, Missouri, USA)			6.00
Lipase from porcine pancreas (Sigma-Aldrich, St. Louis, Missouri, USA)			1.00
Bile (Sigma-Aldrich, St. Louis, Missouri, USA)			10.00

Table S2. Primer sequences for housekeeping genes and genes of interest for qPCR

GENE	NAME	FW	RV	MELTING T
**IL-1β**	Interleukin 1β	AGA TGA TAA GCC CAC TCT ACA G	ACA TTC AGC ACA GGA CTC TC	58/60
**NF-kB**	Nuclear factor kappa-light-chain-enhancer of activated B cells	ACA GCT GGA TGT GTG ACT GG	TCC TCC GAA GCT GGA CAA AC	60
**IL-8**	Interleukin 8	GGA CAA GAG CCA GGA AGA AA	CCT ACA ACA GAC CCA CAC AAT A	58/60
**ZO-1**	Zonula Occludens-1	TTC ACG CAG TTA CGA GCA AG	TTG GTG TTT GAA GGC AGA GC	58

**OCCLUDIN**	Occludin	GGG CAT TGC TCA TCC TGA AG	GCC TGT AAG GAG GTG GAC TT	60
**CLAUDIN-1**	Claudin-1	TGG TCA GGC TCT CTT CAC TG	TTG GAT AGG GCC TTG GTG TT	60/58
**SOD1**	Superoxide dismutase 1	GGTCCTCACTTTAATCCTCTATCCAG	CCAACATGCCTCTCTTCATCC	60
**SOD2**	Superoxide dismutase 2	AGCCCAGATAGCTCTTCAGC	CCAGCAACTCCCCTTTGGG	60
**BACT**	B-Actina	GAA GAT CAA GAT CAT TGC TC	ATC CAC ATC TGC TGG AAG G	61/57
